# Supplementary material for: An ELIXIR scoping review on domain-specific evaluation metrics for synthetic data in life sciences
Source: NAR Genom Bioinform. 2026 Feb 11;8(1):lqag012. doi: 10.1093/nargab/lqag012 (PMC12891913; doi:10.1093/nargab/lqag012)
Supplement: lqag012_Supplemental_Files [file lqag012_supplemental_files.zip › Supplementary Material.pdf]

Supplementary Material for

An ELIXIR Scoping Review on Domain-Specific Evaluation Metrics for Synthetic Data in Life Sciences

Styliani-Christina Fragkouli [1,2], Somya Iqbal [3], Lisa Crossman [4,5], Barbara Gravel [6,7,8], Nagat Masued [9], Mark Onders [10], Devesh Haseja [11], Alex Stikkelman [12], Alfonso Valencia [9,13], Tom Lenaerts [6,7,8], Fotis Psomopoulos [2], Pilib Ó Broin [11], Núria Queralt-Rosinach [12], Davide Cirillo[9]

Queries and PRISMA diagrams

This section presents the specific search queries developed for different types of synthetic data (SD) to ensure comprehensive coverage of the topic. Each search strategy was tailored to account for the nuanced terminology of its respective domain and adjusted to incorporate database-specific filters, such as Open Access restrictions and subject area classifications. This multi-layered approach supports a robust and thorough review of the current literature, highlighting trends and identifying gaps across SD applications over the past decade. Corresponding PRISMA flow diagrams for each domain are provided in Supplementary Figures S1–S6.

Synthetic Genomics

PubMed: ("simulated NGS" OR "synthetic NGS" OR "simulated sequencing data") AND ("evaluation") - 74 articles

SCOPUS: TITLE-ABS-KEY (("simulated NGS" OR "synthetic NGS" OR "simulated sequencing data") AND ("quality" OR "evaluation" OR "performance" OR "assessment")) AND LIMIT-TO (OA, "all") AND LIMIT-TO (SUBJAREA, "MEDI")

Google Scholar: ("simulated NGS" OR "synthetic NGS" OR "simulated sequencing data") AND ("quality metrics" OR "performance metrics" OR "evaluation metrics" OR "assessment metrics")

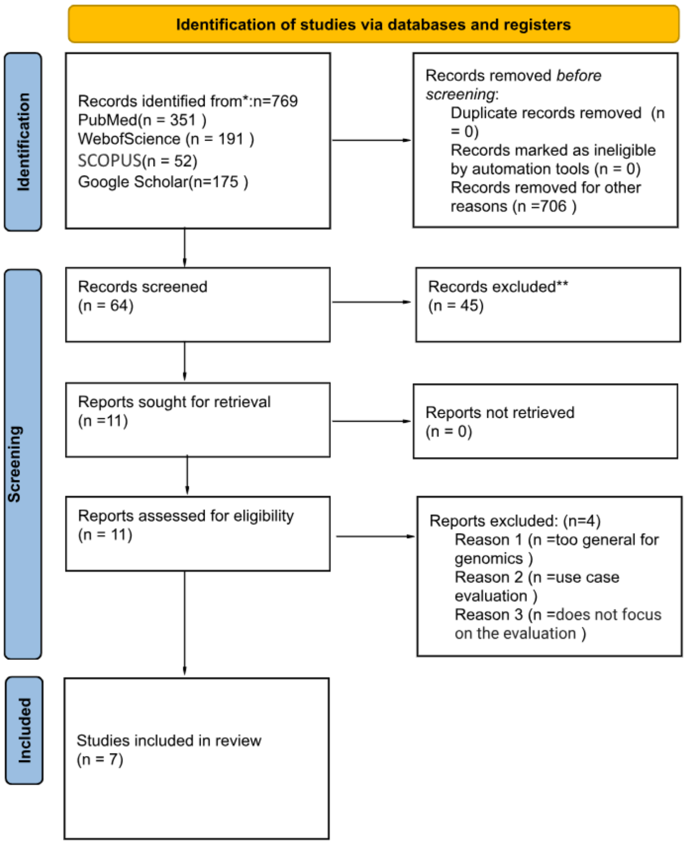

Fig. S1: The PRISMA flow chart for the genomics domain.

Synthetic Transcriptomics

- ("synthetic data generation" OR "synthetic data" OR "data generation") AND ("transcriptome" OR "transcriptomic" OR "gene expression" OR "RNA-sequencing" OR "RNA-seq" OR "single-cell RNA" OR "scRNA-seq") AND ("deep learning" OR "machine learning") OR ("metric")

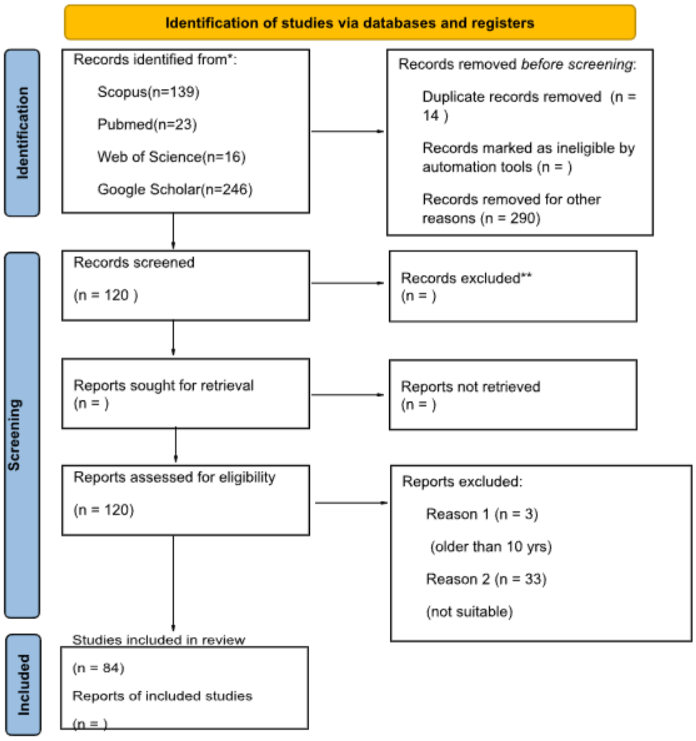

Fig. S2: The PRISMA flow chart for the transcriptomics domain.

Synthetic Proteomics

PubMed: ("synthetic data" AND "proteomics") - 14 results
- (("synthetic" AND ("proteomics" OR "proteomics data")) AND (("quality" OR "evaluation" OR "performance" OR "assessment") AND "metrics")) - 5 results
- ("proteomics" AND "synthetic data") - 33 results

SCOPUS: TITLE-ABS-KEY (proteomics) AND TITLE-ABS-KEY ("synthetic data") - 31 results

Google Scholar:
- "SYNTHETIC PROTEOMIC DATA AND "metrics" OR "data quality" "synthetic proteomic data"" - 4 results
- "synthetic data AND proteomics AND "quality" OR "metrics" OR "data evaluation"" - 424,000 results, sorted by relevance, top 20 selected

Web of Science:
- Topic=("proteomics" AND "synthetic data")
- Abstract=("synthetic data" AND "proteomics") AND Timespan: 2013-01-01 to 2023-12-30 - 16 results

DOAJ.org: "Proteomics AND "synthetic data"" - 6 articles

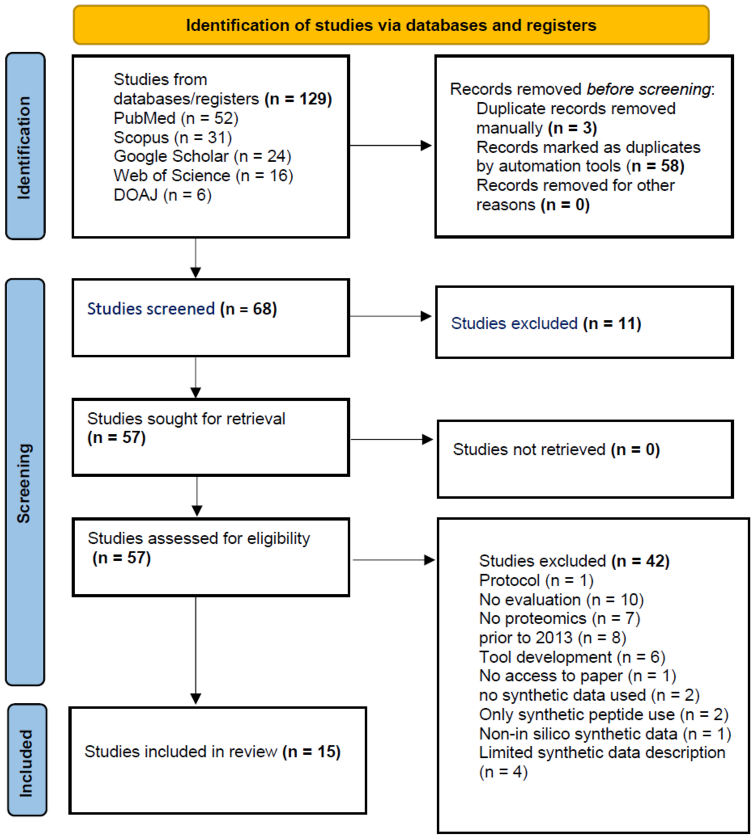

Fig. S3: The PRISMA flow chart for the proteomics domain.

Synthetic Phenomics

PubMed: ("synthetic data" AND "phenomics") AND (2012/1/1:2013/4/20[pdat]) - 2 results

Google Scholar:
- ALL words=("synthetic data" AND phenomics AND "quality metrics") AND Full text=("synthetic data AND phenomics") AND Timespan: 2012-2023 - 12 results
- ALL words=("synthetic data" AND phenomics) FULL TEXT=("synthetic data AND phenomics) Timespan: 2012-2023 - 6890 results

Web of Science:
- ALL=("synthetic data" AND "phenomics") AND Timespan: 2012-01-01 to 2023-04-20 - 21 results
- ALL=("synthetic" AND "phenomics" AND evaluat*) AND Timespan: 2012-01-01 to 2023-04-20 - 7 results
- ALL=("synthetic data" AND "phenomics" AND evaluat*) AND Timespan: 2012-01-01 to 2023-04-20 - 5 results

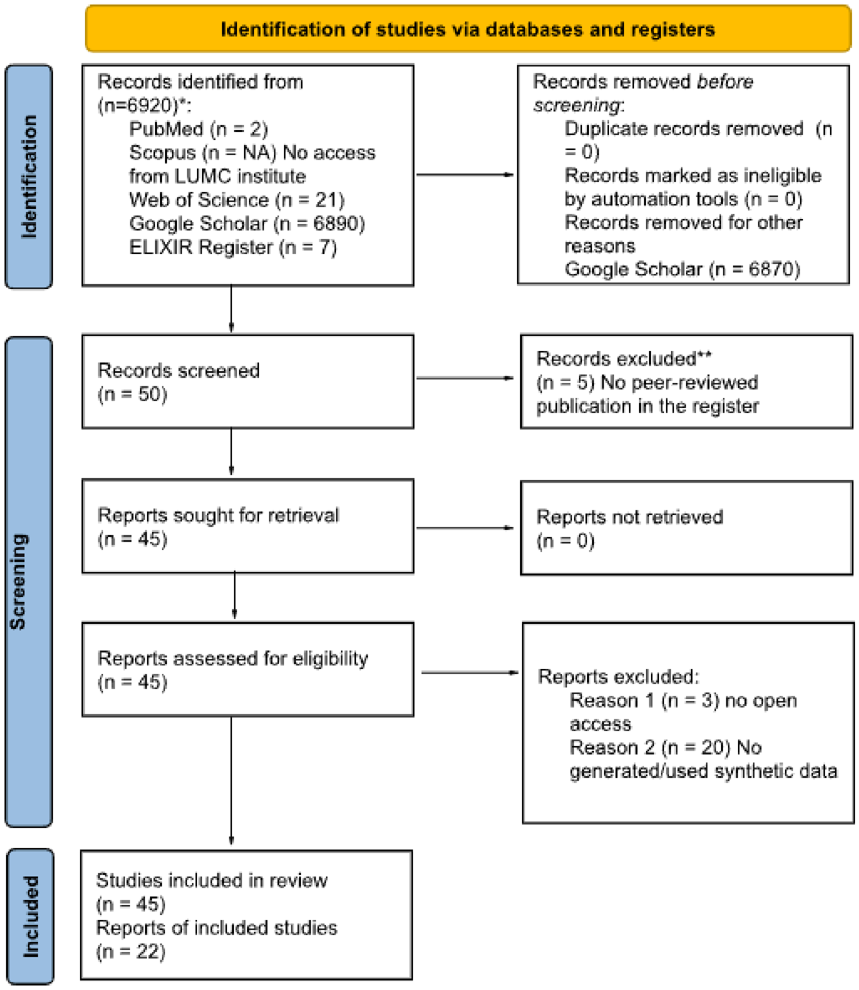

Fig. S4: The PRISMA flow chart for the phenomics domain.

Synthetic Imaging

PubMed: ("synthetic images" AND ("quality metrics" OR "performance metrics" OR "evaluation metrics" OR "assessment metrics")) - 2 Open Access + 8 not free

SCOPUS: TITLE-ABS-KEY ("synthetic images" AND (("quality" OR "evaluation" OR "performance" OR "assessment") AND "metrics")) AND LIMIT-TO (OA, "all") AND LIMIT-TO (SUBJAREA, "MEDI") - 17 Open Access

Google Scholar: "synthetic medical images" AND ("quality metrics" OR "performance metrics" OR "evaluation metrics" OR "assessment metrics") - 244 results

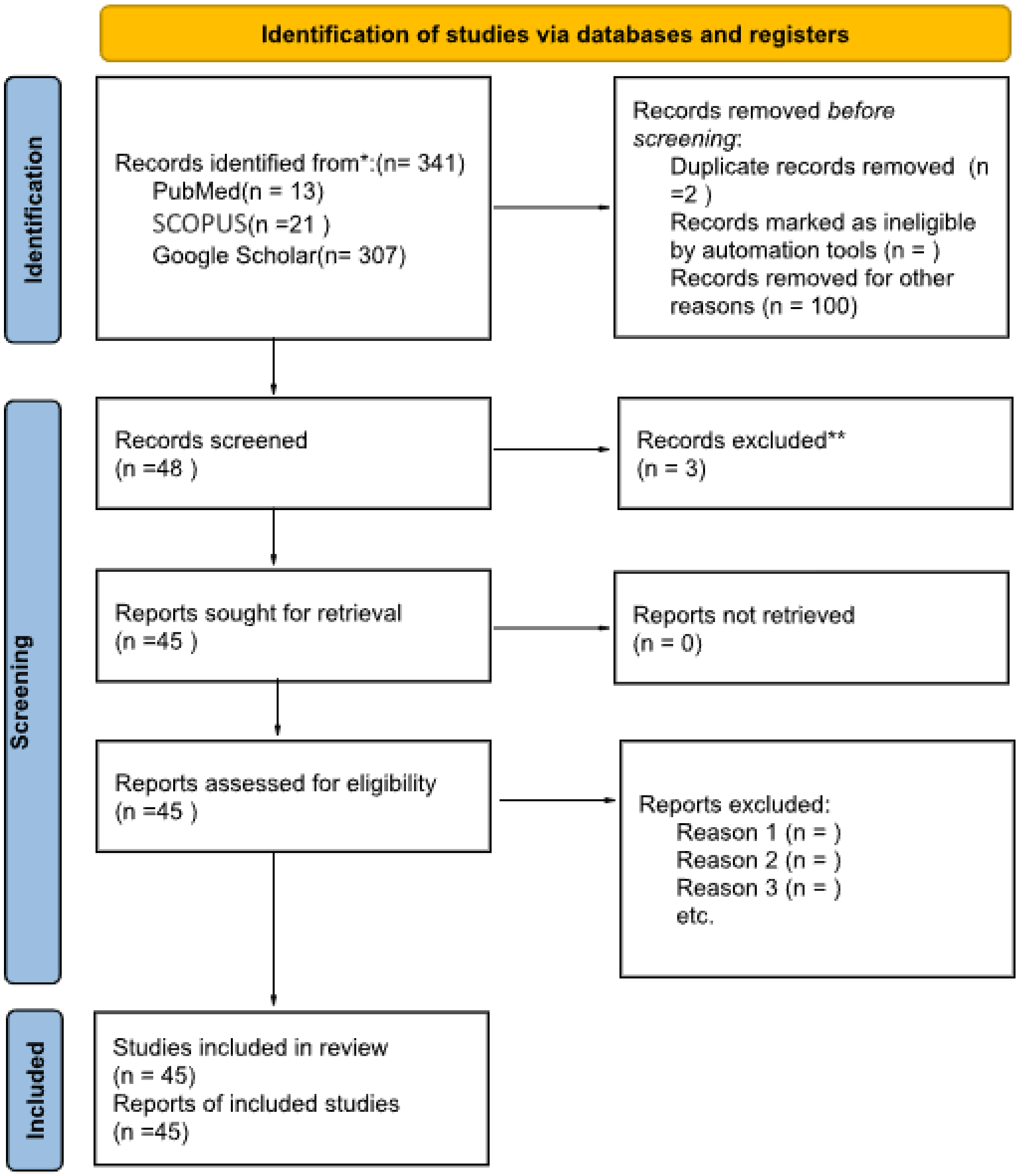

Fig. S5: The PRISMA flow chart for the medical imaging domain.

Synthetic Electronic Health Records

PubMed: (("synthetic" AND ("Electronic health records" OR "Electronic health data" OR "Tabular patient records" OR "Tabular patient data")) AND (("quality" OR "evaluation" OR "performance" OR "assessment") AND "metrics")) - 12 results

SCOPUS: TITLE-ABS-KEY (("synthetic" AND ("Electronic health records" OR "Electronic health data" OR "Tabular patient records" OR "Tabular patient data")) AND (("quality" OR "evaluation" OR "performance" OR "assessment") AND "metrics")) - 18 results

Google Scholar: "synthetic electronic health records" AND ("quality metrics" OR "performance metrics" OR "evaluation metrics" OR "assessment metrics") - 54 results

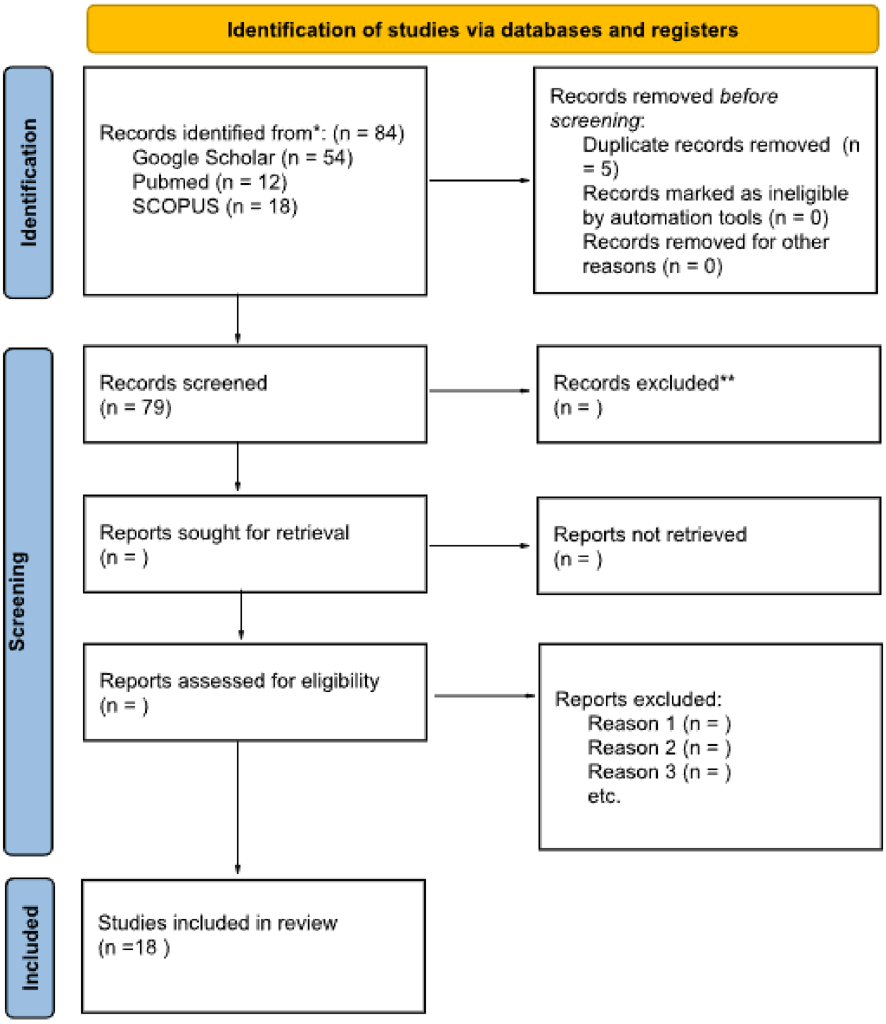

Fig. S6: The PRISMA flow chart for the EHR domain.

Detailed list of quality metrics identified for proteomics

Quantitative metrics (including statistical and performance):

- Signal-to-Noise Ratio (SNR) - image quality metric expressed in dB (image based SD), Spot Efficiency - evaluating spot detection performance (image based SD). FDR (true spots versus false - image based SD), Subtraction index with background pixels, Sensitivity, true spots detected divided by total true spots. (image based SD), Low-abundance Protein Detection (LPD), percentage of low-abundance spots detected. Other ML related metrics.
- Gaussian-distributed Perturbation, Stepwise perturbations, simulating calibration uncertainty in peak fitting, Modelling of acquisition windows, noise and collision based classifications (MS), Standard Deviation of Gaussian fit - measuring precision in peak intensity fitting, Normalized Separation Parameter ($\chi = dt / HWHM$) - for peak separation analysis.
- True Positive Distance Threshold - peak is a true positive if within 0.3% m/z of true peak, Cosine Similarity Threshold - isotope pattern matching threshold, thresholding variations in expression, Manhattan Distance threshold - peptide matching threshold set at 10 units.
- Noise Levels / Perturbation Levels - evaluated as factors influencing AUC, sensitivity, and specificity.
- Protein expression levels and effect sizes (modulation to simulate RD)
- AUC (area under the curve), ROC (receiver operating characteristic curve) - model efficacy
- Poisson-distributed Error Modeling - simulating counting errors with variance proportional to sqrt(N).
- Feature matrix - Alignment, peak binning, peak detection, Randomised noise for predictor features, exponential noise
- F1 Score - harmonic mean of (1 - FDR) and Sensitivity.
- ROC Curve - showing TPR (True Positive Rate) vs. FPR (False Positive Rate).
- MSPE (Mean Square Prediction Error) - for dose-response model comparison.
- Data distributions: Binomial distribution for feature clusters, Assortativity distributions (networks), Random sampling of distributions, Markov chain Monte Carlo (MCMC)
- False Discovery Rate (FDR) - false spots divided by total spots detected.
- BSI - pre-processing performance measure.
- Recall (Recovery Rate) - fraction of recaptured initial seed nodes or disease proteins.
- Precision - true positives divided by (true positives + false positives).
- MAE (Mean Absolute Error) - used in network noise reduction performance.
- BH-adjusted P-value - used to assess model classification significance (cutoff < 0.05).
- Canonical coefficients related to SD weights
- Balancing of datasets during evaluation of models, replicate consistency, batch effects

Qualitative Metrics:

- Subjective (Perceptual) Evaluation - visual assessment of image fidelity to real 2DGE images.
- Visual data distribution graphs and counts between RD and SD
- Generating replicates - similarity
- Manual inspection of peptides and lengths (amino acids)

- network connectivity and node size to simulate RD
- Use of ground truth labels

Biological and domain specific Metrics:

- SNR - capturing heterogeneity in expression data
- Protein abundance values/ranges, peptide lengths, isotope pattern fidelity
- Pooling for minimising isobaric peptide signals
- representation of disease associated proteins (% known, subsets), module overlaps
- Ground truth with biological sets.
- Similarity (true molecular ions)

Comparison metrics:

- Overlap Percentage - comparing model predictions with seed perturbation scenarios.
- Number of Oscillating Proteins Recovered - metric from MOSAIC modeling.
- Comparing model performance on RD and SD (characteristics and features specific to study context) - PSM, identifications, counts correct number of classifications).
- Graphed data comparisons, distributions, counts and relative differences under varying conditions (perturbations, noise, thresholds).

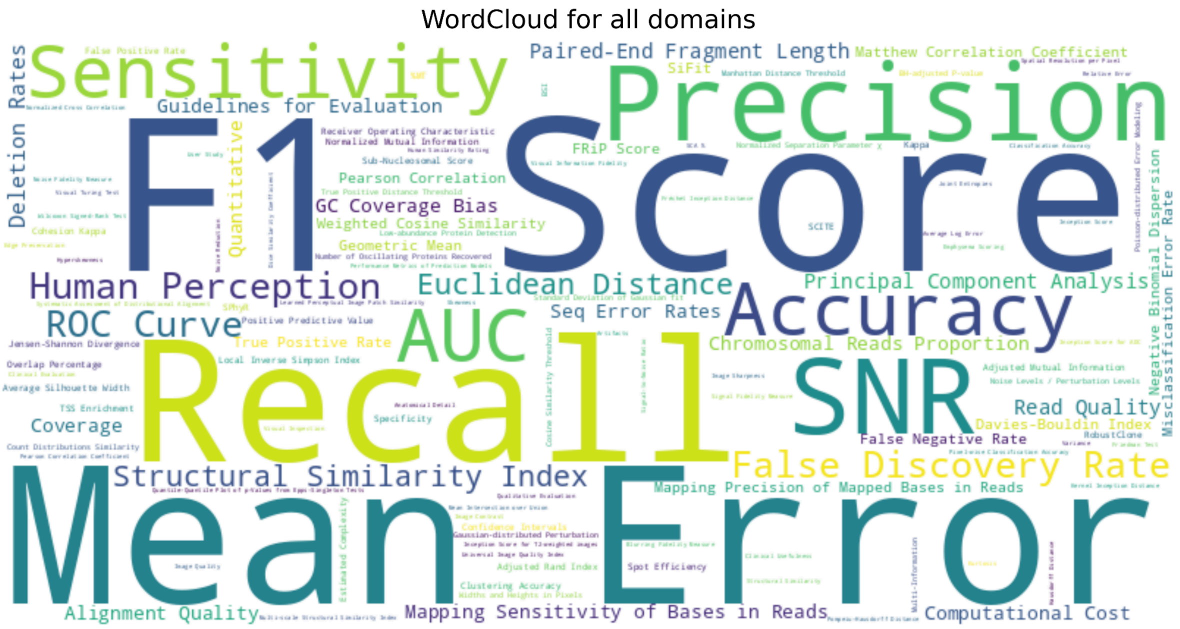

Fig. S7. Word cloud visualization of the metrics identified across the six domains: genomics, transcriptomics, proteomics, phenomics, imaging and EHRs.

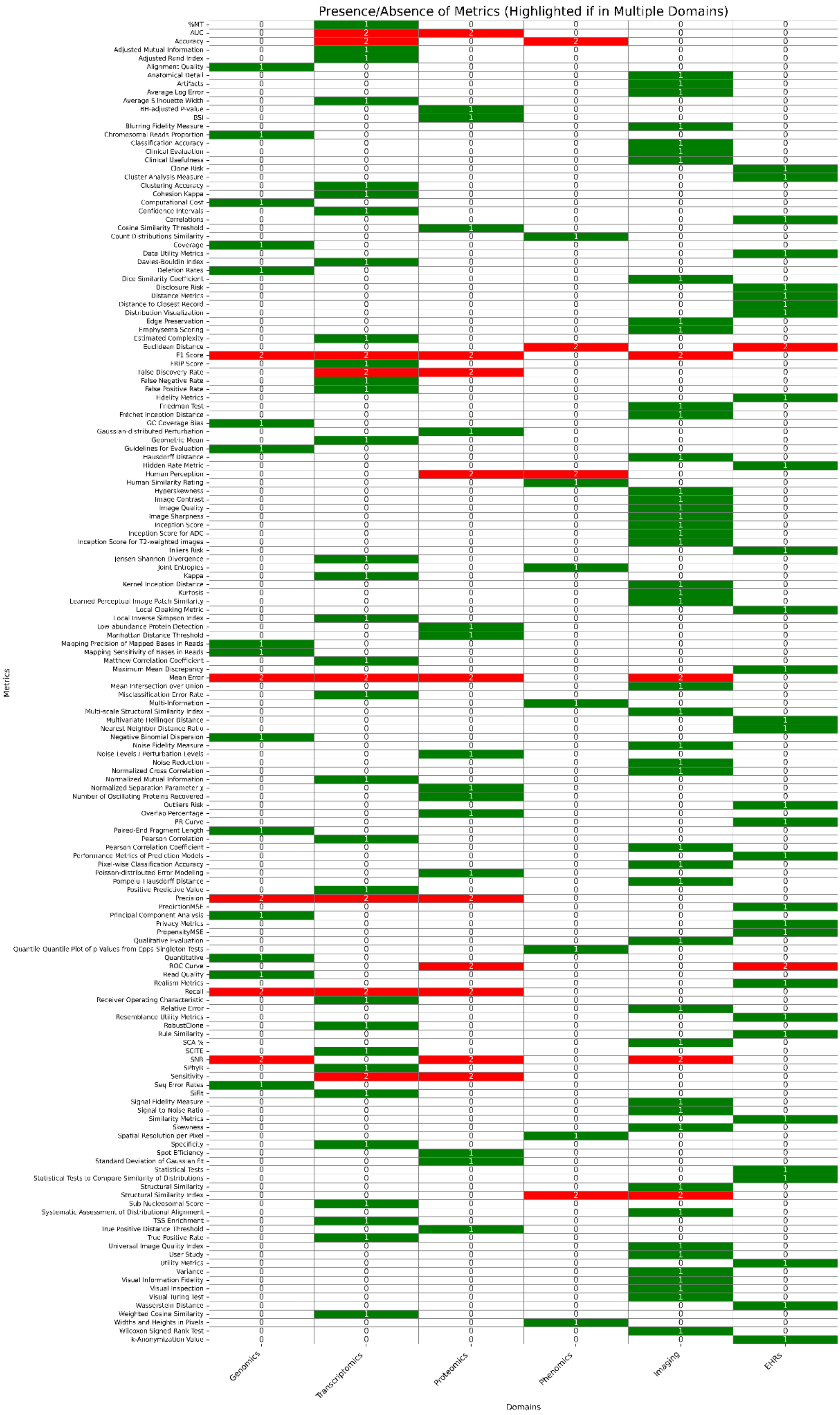
Presence/Absence of Metrics (Highlighted if in Multiple Domains)
Metrics
%MT
AUC
Accuracy
Adjusted Mutual Information
Adjusted Rand Index
Alignment Quality
Anatomical Detail
Artifacts
Average Log Error
Average Silhouette Width
BH-adjusted P-value
BSI
Blurring Fidelity Measure
Chromosomal Reads Proportion
Classification Accuracy
Clinical Evaluation
Clinical Usefulness
Clone Risk
Cluster Analysis Measure
Clustering Accuracy
Cohesion Kappa
Computational Cost
Confidence Intervals
Correlations
Cosine Similarity Threshold
Count Distributions Similarity
Coverage
Data Utility Metrics
Davies-Bouldin Index
Deletion Rates
Dice Similarity Coefficient
Disclosure Risk
Distance Metrics
Distance to Closest Record
Distribution Visualization
Edge Preservation
Emphysema Scoring
Estimated Complexity
Euclidean Distance
F1 Score
FRiP Score
False Discovery Rate
False Negative Rate
False Positive Rate
Fidelity Metrics
Friedman Test
Fréchet Inception Distance
GC Coverage Bias
Gaussian-distributed Perturbation
Geometric Mean
Guidelines for Evaluation
Hausdorff Distance
Hidden Rate Metric
Human Perception
Human Similarity Rating
Hyperskewness
Image Contrast
Image Quality
Image Sharpness
Inception Score
Inception Score for ADC
Inception Score for T2-weighted images
Inliers Risk
Jensen-Shannon Divergence
Joint Entropies
Kappa
Kernel Inception Distance
Kurtosis
Learned Perceptual Image Patch Similarity
Local Cloaking Metric
Local Inverse Simpson Index
Low-abundance Protein Detection
Manhattan Distance Threshold
Mapping Precision of Mapped Bases in Reads
Mapping Sensitivity of Bases in Reads
Matthew Correlation Coefficient
Maximum Mean Discrepancy
Mean Error
Mean Intersection over Union
Misclassification Error Rate
Multi-Information
Multi-scale Structural Similarity Index
Multivariate Hellinger Distance
Nearest Neighbor Distance Ratio
Negative Binomial Dispersion
Noise Fidelity Measure
Noise Levels / Perturbation Levels
Noise Reduction
Normalized Cross Correlation
Normalized Mutual Information
Normalized Separation Parameter χ
Number of Oscillating Proteins Recovered
Outliers Risk
Overlap Percentage
PR Curve
Paired-End Fragment Length
Pearson Correlation
Pearson Correlation Coefficient
Performance Metrics of Prediction Models
Pixel-wise Classification Accuracy
Poisson-distributed Error Modeling
Pompeiu-Hausdorff Distance
Positive Predictive Value
Precision
PredictionMSE
Principal Component Analysis
Privacy Metrics
PropensityMSE
Qualitative Evaluation
Quantile Quantile Plot of p Values from Epps Singleton Tests
Quantitative
ROC Curve
Read Quality
Realism Metrics
Recall
Receiver Operating Characteristic
Relative Error
Resemblance Utility Metrics
RobustClone
Rule Similarity
SCA %
SCITE
SNR
SPhyR
Sensitivity
Seq Error Rates
SiFit
Signal Fidelity Measure
Signal to Noise Ratio
Similarity Metrics
Skewness
Spatial Resolution per Pixel
Specificity
Spot Efficiency
Standard Deviation of Gaussian fit
Statistical Tests
Statistical Tests to Compare Similarity of Distributions
Structural Similarity
Structural Similarity Index
Sub Nucleosomal Score
Systematic Assessment of Distributional Alignment
TSS Enrichment
True Positive Distance Threshold
True Positive Rate
Universal Image Quality Index
User Study
Utility Metrics
Variance
Visual Information Fidelity
Visual Inspection
Visual Turing Test
Wasserstein Distance
Weighted Cosine Similarity
Widths and Heights in Pixels
Wilcoxon Signed Rank Test
k-Anonymization Value
Genomics
Transcriptomics
Proteomics
Phenomics
Imaging
EHRs
Domains

Fig. S8: A heatmap that displays the presence or absence of various metrics across multiple domains. Green cells indicate that the metric is uniquely present in that domain and red cells denote metrics that are shared among multiple domains. This color coding allows for a quick visual assessment of which metrics are domain-specific and which are common across domains.
